# The Association Between Short-Chain Fatty Acids and the Incidence of Food Allergies—Systematic Review

**DOI:** 10.3390/nu17193117

**Published:** 2025-09-30

**Authors:** Iga Szukalska, Maciej Ziętek, Jacek Brodowski, Małgorzata Szczuko

**Affiliations:** 1 Department of Bromatology and Nutritional Diagnostics, Pomeranian Medical University in Szczecin, 71-460 Szczecin, Poland; 2 Department of General Pharmacology and Pharmacoeconomics, Pomeranian Medical University in Szczecin, 71-460 Szczecin, Poland; maciej.zietek@pum.edu.pl; 3 Department of Perinatology, Obstetrics and Gynecology, Pomeranian Medical University in Szczecin, 70-204 Szczecin, Poland; 4 Primary Care Department, Pomeranian Medical University in Szczecin, Żołnierska 48, 71-210 Szczecin, Poland; jacek.brodowski@pum.edu.pl

**Keywords:** allergy, SCFA, acetic acid, propionic acid, butyric acid, 3,4-methylvaleric acid

## Abstract

Background/Objectives: There is a constant increase in the incidence of food allergies. Short-chain fatty acids (SCFAs), responsible for maintaining the intestinal barrier and immune balance, are produced by the intestinal microbiota through the fermentation of dietary fibre. The aim of this systematic literature review was to analyse the association of SCFAs with the occurrence of food allergies. Methods: The analysed data were obtained by searching the PubMed and Scopus databases with the keywords “short-chain fatty acids and allergy”, “SCFA and allergy”, “SCFA and food allergy”, and “acetic acid or propionic acid or butyric acid or 3,4-methylvaleric acid and allergy”. Exclusion criteria were as follows: retracted articles, articles not freely accessible, and incomplete/insufficient data (conference reports). Two authors independently searched the literature. Results: Patients with food allergies show lower levels of SCFAs, especially acetic, butyric and propionic acids. 3,4-methylvaleric acid may be associated with intestinal inflammation in infants and intestinal leakage. Conclusions: Based on studies, there appears to be an association between lower stool levels of SCFAs—particularly butyric, propionic, acetic, and isovaleric acids—and the occurrence of food allergies in both children and adults. A proper diet that strengthens fibre, probiotics, and prebiotics and limits antibiotics, xenobiotics, pesticides is crucial for maintaining adequate SCFA levels and thus reducing allergy-related symptoms. 3,4-Methylvaleric acid and the activation of inflammatory processes contributing to intestinal leakage may provide a new diagnostic path.

## 1. Introduction

### 1.1. Overview of Characteristics of Short-Chain Fatty Acids (SCFAs)

The gut microbiota constitutes one of the main sources of short-chain fatty acids (SCFAs), which are produced through the fermentation of dietary fibre and carbohydrates supplied in the diet. SCFAs are made up of short carbon chains containing 2–6 carbon atoms [1]. In the human body, the main site of SCFA production is the colon, where intestinal bacteria can reach a total concentration of 50–150 mM [2]. SCFAs are mainly formed from acids such as formic, acetic, propionic, butyric, valerian, and caproic acids [3]. The level and diversity of SCFAs vary throughout an organism’s life. Initially, acetic acid is the dominant SCFA, but over time, as the organism continues to develop, the diversity of the gut microbiota and its products is shaped by a number of factors like pH, diet, xenobiotics, health condition and medications taken [1]. One such factor is the pH of the gut; in the proximal part, the pH is around 5.6, which favours the formation of propionic and butyric acids. In contrast, conditions further down in the gut, specifically a pH value of around 6.3, favour fermentation by *Bacteroides* [2].

In the human intestinal microbiome, there are two predominant types of bacteria: *Bacteroidota* (*Bacteroidetes*) and *Bacillota* (*Firmicutes*). The *Bacteroides*, *Prevotella*, *Parabacteroides*, and *Alistipes* types have a particular ability to break down complex carbohydrates thanks to specialised enzymes known as CAZymes. On the other hand, bacteria of the *Firmicutes* type, for example, *Faecalibacterium prausnitzii*, *Eubacterium rectale*, and *Eubacterium hallii*, are specialised in degrading dietary fibre via the enzyme butyryl-coenzyme A, resulting in butyric acid [4]. In the *Verrucomicrobia phylum*, recently gaining in relevance, *Akkermansia muciniphila* also produces propionate [2].

### 1.2. Brief Characteristics of Branched-Chain Fatty Acids (BCFAs)

Intestinal bacteria ferment proteins and amino acids, resulting in the production of SCFAs and branched-chain fatty acids (BCFAs) [2]. High-protein diets lead to increased levels of BCFAs in the intestines [5], partly because branched-chain amino acids (BCAAs) such as valine, leucine, and isoleucine are fermented by intestinal bacteria into BSCFAs [6]. It has been found that morbid obesity may reflect a slower conversion of BCAAs to BCFAs [3]. Among BCFAs, we can distinguish two groups: monomethyl and polymethyl BCFAs. Monomethyl BCFAs can be divided into long-chain branched-chain monomethyl fatty acids (BLCFAs) and short-chain branched-chain monomethyl fatty acids (BSCFAs) [5]. Examples of BCFAs include isovalerate, 2-methylbutyric, and isobutyric. Their concentrations are much lower than those of SCFAs [2], and they are mainly found in the gut. Blood levels of BSCFAs are very low [7]. In infants, until the gut microbiota is more complex, they are not produced in significant amounts [3]. BCFAs influence many signalling pathways. Studies reveal potent anticancer, lipid-lowering, anti-inflammatory, and neuroprotective effects [7]. However, our knowledge of the effects of individual SCFAs remains limited. Isobutyric acid has been shown to have the strongest effect among BSCFAs on both immune system activity and tumour growth [8]. Isocaproic acid, on the other hand, was selected as a non-specific marker of Clostridium difficle in the gut microbiota of young children [9].

### 1.3. Mechanism of Action of Short-Chain Fatty Acids

SCFAs provide a source of energy for colonocytes but can also be transported into the bloodstream, affecting the body’s metabolism [10]. SCFAs affect the immune system and modulate the intestinal epithelium through different mechanisms. One way in which SCFAs act in the body is through G-protein-coupled receptors (GCPRs), which are found in intestinal cells and various other cells in the body, such as dendritic cells and regulatory T cells (Tregs) [11]. These receptors show specificity to individual SCFAs based on their chain length. For example, GPR43, located on immune cells, has a greater affinity for shorter SCFAs, such as acetate and propionate [4]. Another example is the GCPR1 receptor, which binds more weakly to acetic acid but more strongly to propionic, butyric acids, and valeric acid [12]. Butyric acid induced activation of GPR109a results in an increased tolerogenic macrophage response and induces intestinal homeostasis by reducing colonic inflammation [2].

SCFAs, in combination with receptor-mediated effects, can even exert effects at the epigenetic level. One of their known actions is the inhibition of histone deacetylases, but also the alteration of histones during histone propionylation and butyrylation [13]. Specifically, propionic and butyric acids cause an increase in IL-10 expression in lymphocytes through histone deacetylase (HDAC) inhibition. Additionally, it has been noted that SCFAs activate innate immunity. In this process, SCFAs are used as precursors of acyl-CoA, thereby directly transferring carbon atoms to histones via metabolic–epigenetic linkages, causing histone acetylation via histone acetyltransferase (HAT). Therefore, SCFAs are thought to provide the material needed to form acetyl-CoA and other endogenous metabolites in immune cells and intestinal epithelial cells [14]. Another way SCFAs exert their effects is through their uptake by intestinal epithelial cells from the intestinal lumen. The main energy source in the mitochondria is the β-oxidation of fatty acids, including butyric acid. This process provides some of the highest levels of oxygen consumed by human colonocytes. Other SCFAs are also metabolised. Acetate, for example, is metabolised in the muscle and is a substrate for cholesterol and fats. In contrast, propionate is the precursor for glucose synthesis in the liver [2]. SCFAs reach the liver via the portal vein and can therefore regulate metabolism and energy homeostasis [9].

Studies of immunoglobulin E (IgE)-dependent and IgE-independent food allergies have shown that SCFAs play an essential role in regulating the immune response. Their action also reduces the incidence of histamine reactions and inhibits the release of inflammatory mediators. The role of mast cells is to initiate and then maintain inflammation, and their repeated exposure to an allergen induces the aggregation of FcεRI by IgE on the cell membrane. This condition causes rapid mast cell degranulation, which triggers the release of multiple inflammatory mediators, followed by the production of inflammatory cytokines such as tumour necrosis factor alpha (TNFα) and interleukin 6 (IL-6). In contrast, studies have shown that butyric acid inhibits mast cell function in an allergen-induced airway obstruction model [15]. It also inhibits allergen-induced histamine release, which may be of relevance to those affected by food allergies [14]. Short-chain fatty acids affect B lymphocytes, which use SCFAs as activators for active immune responses. They cause an increase in IgG and IgA production, which contributes to enhanced humoral immunity and an increase in IgA-coated bacteria in the gut. This action has the effect of preventing dysbiosis and regulating the composition of microorganisms in the gut [16].

Short-chain fatty acids (SCFAs), through their pleiotropic roles in metabolic regulation, modulation of inflammatory pathways, and mediation of gut–brain signalling, constitute a promising focus for elucidating mechanisms contributing to the pathogenesis of non-communicable diseases. The limited number of studies addressing the impact of SCFAs on human immunology and allergy development highlights the need for further investigation in this field. The aim of this study is to evaluate how short-chain fatty acids modulate immune responses and whether their profile may serve as a biomarker of allergy risk or a potential target for dietary interventions.

## 2. Material and Methods

Studies for systematic review were identified through a systematic search of databases and registries using PRISMA guidelines [17]. In this study on the association of SCFAs with the occurrence of food allergies, the literature from the last 10 years, obtained from the PubMed, ScienceDirect and Scopus databases, was reviewed. All articles were qualified based on the abstract. The following keywords were used in the search: “short-chain fatty acids and allergy”. “SCFA and allergy,” “SCFA and food allergy,” “butyric acid and allergy,” “acetic acid and allergy,” “propionic acid and allergy,” “branched-chain fatty acids and allergy” published by the end of June 2025.

**Figure 1:**
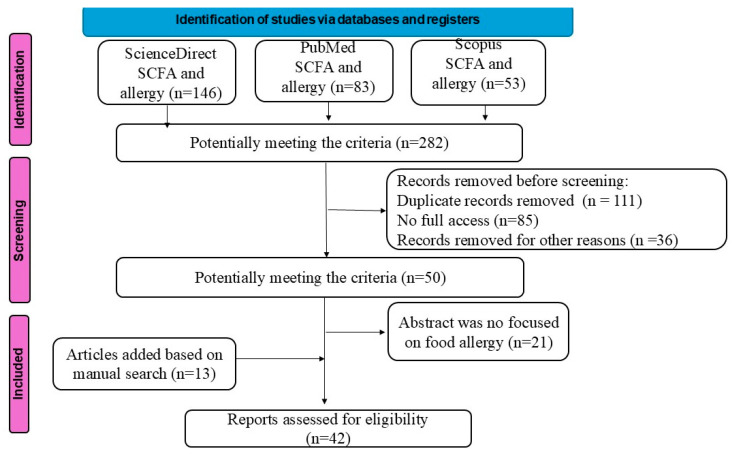
PRISMA flow diagram.

Relevant articles and reviews examining the relationship between SCFAs and the occurrence of allergy symptoms were included. Similar articles, as suggested by the databases, were also searched for inclusion. References to all relevant publications and systematic reviews identified during the initial search were also searched. Exclusion criteria were as follows: retracted articles, articles not freely accessible, and incomplete/insufficient data (conference reports). Two authors independently searched the literature and, after eliminating duplicate articles, assessed their eligibility according to the above criteria. The method of searching for data is presented below (Figure 1). The studies tabulating the study intervention characteristics are presented in Table 1 and Table 2.

## 3. Results

### 3.1. Factors Influencing SCFA Levels

One factor influencing SCFA levels is diet. This factor, quite broadly defined, can affect the person consuming it in several ways. Firstly, the composition of the diet, specifically the type, level, and plant source of fermentable dietary fibre, can influence the composition and level of SCFAs in the body. One way is through the expression of active carbohydrate enzymes, also known as CAZymes, caused by fibre-degrading bacterial species [23]. The Western diet, which is quite common in modern society, is low in fibre but high in refined carbohydrates. Long-term consumption of the Western diet may result in changes in the host microbiota, manifesting in a decrease in its diversity and a subsequent change in the substrates it uses. It is therefore important to follow fibre-rich diets to counteract these effects and enrich the SCFA pool. Constituents that positively influence butyric acid formation are starch, whole grains, and bran in cereals containing arabinoxylans [24]. Arabinoxylans are polysaccharides with a heterogeneous structure and are components of dietary fibre. They are found in foods such as oats, rye, wheat, and maize [24]. The effects of the dietary intake of arabinoxylan in the body include the quantitative enrichment of microorganism diversity, for example, *Faecalibacterium pausnitzii* or *Roseburia intestinalis*, as shown in studies conducted on pigs, which contributes to an increase in butyric acid levels. In contrast, a diet enriched with resistant starch plays less of a role in increasing butyric acid levels, but it is still better than a Western diet. Studies in humans have shown an increase in *Bifidobacterium*, which produces acetate that can be converted to butyric acid [23].

Another component of dietary fibre is pectin, which has the ability to modulate the ratio of *Firmicutes* to *Bacteroidetes bacteria*, resulting in an increase in the concentration of SCFAs in both faeces and serum [25]. Microorganisms also use dietary amino acids to produce SCFAs. One of the exogenous amino acids supplied in the diet is threonine, which is metabolised into the main SCFAs (propionic and butyric acid) [26]. Food products that supply it through the diet include meat and fish [16]. In addition, the beneficial effects of faecal microbiota transplantation, a more invasive procedure that can alter the composition of the recipient’s gut microbiome, have been demonstrated [11]. Another factor that negatively impacts the formation of short-chain fatty acids is dysbiosis in the host. Many aspects can contribute to its development, but the main ones are the aforementioned poor diet, antibiotic use, lack of breastfeeding, mode of delivery, and other environmental factors like pestycydy, herbicydy, plastiki (mikroplastik, BPA, ftalany) [11,25].

The intake of xenobiotics also has an adverse effect on the composition of the microbiome. Xenobiotics are a group of chemical substances, the most well-known of which are pesticides. They not only disrupt the composition of the microbiota but also have a toxic or even carcinogenic effect on the body [27]. Based on research, it can be hypothesised that the gut microbiota begins to develop during the foetal stage, as the mother’s SCFA levels affect both her and her unborn child [28]. Therefore, it is believed that SCFAs have an impact on the immune development of the unborn child from the moment of conception. One of the described approaches to beneficially modulating the microbiome is the inclusion of supplements containing prebiotic fibre in the mother’s diet. Prebiotic fibre is characterised by its protective and beneficial effects on the body. Examples of prebiotic fibre include fructooligosaccharides (FOS) and galactooligosaccharides (GOS), which are highly desirable in the diet. These substances play a positive role in the growth of commensal bacteria, especially *Bifidobacterium* spp., but they also lower the pH of the intestines. The supplementation of FOS and GOS resulted in an increase in the total level of SCFAs in mothers, especially acetic acid [29]. The way in which the baby is delivered also plays an important role. Newborns born by caesarean section are colonised by *Staphylococcus*, *Enterococcus*, and *Klebsiella* bacteria from the mother’s skin surface or the operating theatre. Newborns born by natural birth show colonisation with bacteria present in the mother’s vaginal flora [27].

The way the baby is fed is another important factor affecting the formation of short-chain fatty acids early in life. Many studies have indicated that breastfeeding has positive effects on the child’s later health. More specifically, breast milk oligosaccharides, abbreviated HMOs, are a solid component of milk. Their specificity of action lies in their minimal digestion in the upper gastrointestinal tract, enabling them to reach the colon in virtually unchanged form. There, they are then utilised by saccharolytic bacteria [29]. Furthermore, the composition of maternal milk, which is also affected by, for example, place of residence, is another important factor in SCFA levels. Another issue that may have an impact is maternal disease, which may contribute to lower SCFA levels in mothers with atopy, which belongs to a broad group of allergic diseases. This condition may affect their offspring later in life because, early in life, the production of SCFAs in the offspring is low because of their immature gut microbiome, which may result in impaired homeostasis in adulthood [30]. The next factor related to the impact of feeding mode in early life is the beneficial fortification of modified milk with prebiotic oligosaccharides when breastfeeding is not possible. The effect observed over time may depend on the type of prebiotic supplement added. To date, supplementation with GOS/FOS or GOS alone has been described as having the best effect. Feeding mixtures to infants enriched with these supplements has been shown to contribute to the development of metabolic activity similar to that of breastfed infants. Supplementation with GOS, playing a bifidogenic role, resulted in an increase in SCFAs, particularly acetic acid. However, the levels of butyric acid and propionic acid were lower in formula-fed infants than in breastfed infants [31].

Another factor influencing SCFA levels is the environment in which one grows up. Growing up on a farm has been associated with higher SCFA levels, attributed to the presence of a more complex gut microbiota. The presence of a pet in the residence is also not without significance. It was found that children living in rural areas with a dog or cat had higher faecal valeric acid concentrations. Having siblings also appears to have a positive effect on SCFA levels, and this effect was independent of rural versus non-rural residence. Higher faecal valerian acid levels were detected in children with older siblings. Therefore, a variety of factors in the environment of growing children can positively contribute to enriching their microbiome [32]. Furthermore, dietary expansion up to 1 year is crucial in the formation of SCFAs. The introduction of yoghurt, fruit, vegetables, and fish into the diet resulted in increased levels of butyric acid. Yoghurt plays a special role in the diet, as butyric acid levels also increase in adults after consuming this product. An adequate diet is a modulating factor for the products formed in the gut [33]. Therefore, it is worth remembering that the diet should be varied, and it is best to choose organically grown and natural products to support a diverse microbiome. Oats, apples, bananas, and asparagus are all good choices because they contain prebiotics and contribute to the growth of *Bifidobacterium* and *Lactobacillus* [27].

The role of supplementation with *Lactobacillus* and *Bifidobacterium* strains in alleviating allergic reactions has been investigated. In an in vitro evaluation, the administration of *Lactobacillus paracasei AH2* isolated from traditional sourdough in China was shown to have the potential to reduce immune reactivity to the wheat protein allergen [34]. In the body, it acts on the pH of the colon, restoring it to the optimal level, as an increase in alkalinity is observed with gluten sensitisation. The provision of *Lactobacillus* paracasei AH2 induces a change in pH, which results in an increase in short-chain fatty acids. One study showed increases specifically in acetate, propionic, butyric acid, and isovaleric through producers such as *Alloprevotella*, *Bacteroides*, and *Faecaliba*. In this study, the results showed a correlation between the production of short-chain fatty acids and Th1/Th2 immune balance and a strengthened intestinal barrier. This condition contributes to a reduction in inflammation, as the intestinal mucosal barrier function is disrupted as a result of a food allergy. This results in increased intestinal permeability to allergens and potentially contributes to intestinal inflammation. The anaphylactic reaction has also been found to be inhibited with higher SCFAs [34].

### 3.2. Effect of SCFAs on Allergy-Related Symptoms

An increasingly common problem affecting millions of people worldwide, especially in industrialised countries, is food allergy. Allergic individuals develop negative symptoms after contact with even small amounts of the food allergen. Clinically, these symptoms manifest as gastrointestinal and respiratory complaints. The main presentations are gastrointestinal disorders, urticaria, and respiratory tract inflammation of varying severity. The most well-known is IgE-dependent food allergy. During this reaction, food-allergen-specific IgE is produced and binds to FcεRIs found on mast cells and basophils, and this occurs right after exposure [35]. This triggers degranulation, i.e., the release of mast cell contents to the extracellular environment. This contributes to the activation of numerous inflammatory mediators and the subsequent production of inflammatory cytokines [15].

According to the hygiene hypothesis, the public health and cultural practices of Western countries reduce the immune stimulation of organisms due to reduced microbial contact with the immune system. These behaviours affect the formation of the gut microbiota and, consequently, the profile of immune system development early in life. A child’s early exposure to the gut microbiota has a beneficial effect on the whole body, as it shifts the Th1/Th2 balance towards Th1. When insufficient stimulation of the immune system occurs, the balance shifts towards Th2, resulting in mediators and the induction of IgE immunoglobulins. This condition results in the maintenance of the allergic response [36].

Human studies have demonstrated that SCFAs are important regulators of the immune response in IgE-dependent and IgE-independent food allergies [15]. The development of food allergies is also associated with a change in mucosal immune tolerance. This may occur as a consequence of an infection or other factors inducing a change in the composition of the intestinal microbiota [35].

Butyric acid, which is a short-chain fatty acid, plays a significant role in inhibiting mast cell activation through various mechanisms. Mast cells are found in body tissues in contact with the external environment. The sites of this contact are the gastrointestinal tract, the respiratory tract, and the skin. In a concentration-dependent manner, butyric acid, but also propionate, has the ability to inhibit IgE/antigen-induced mast cell degranulation. Butyric acid also has the ability to inhibit mast cell activation in response to ionomycin. In addition, butyric acid and propionic acid reduce the secretion of the inflammatory mediator IL-6. The effect of SCFAs on mast cell activity is not dependent on GPR41, GPR43, or PPAR receptors. Another way in which SCFAs can affect mast cells is via the inhibitory effect of butyric acid on HDACs. Butyric acid also acts at the expression level of genes responsible for mast cell activation or cytokine signalling. The presented mechanisms demonstrate the benefits of SCFAs, particularly butyric acid, on mast-cell-dependent allergic disease [15].

One study that measured SCFAs in the plasma of mothers and their infants, as well as in breast milk, showed that lower plasma concentrations of acetic acid, succinic acid, and isobutyric acid in infants were associated with the occurrence of atopic eczema at 12 months of age. This provides confirmation that SCFAs may function as immune modulators [3]. Reduced SCFA levels, often resulting from dysbiosis, may be a risk factor for allergic diseases [11].

Another identified correlation is that high levels of butyric acid are mediated by gut microbes, particularly *Clostridium* spp., which have a protective function against the occurrence of food allergies. SCFAs such as acetate and butyric acid derived from dietary fibre supplied to the body are protective factors against the development of food allergies. The mechanism involves the modification of the DC CD103+ mucosal response. This is considered to be the main cell type responsible for oral tolerance to food antigens by promoting the differentiation of antigen-specific Treg cells. By increasing retinaldehyde dehydrogenase activity in DC CD103+ mucosa, dietary fibre also increases the conversion of vitamin A to retinoic acid, which is necessary for the induction of Treg cells [36]. This mechanism promotes tolerance to food antigens due to the concomitant action of Treg cells reactive to food antigens. Located in the small intestine, Treg cells have the ability to recognise food-derived antigens. In addition, they also inhibit unwanted and negative responses of the body to ingested food, supporting the dominant immune response [9]. Another issue related to food allergies that occurs from infancy may be a mucosal immune imbalance, which can take place as early as immediately after birth. This condition may be influenced by the transmission of the mother’s microbiota composition to the offspring. Therefore, such cases may warrant nutritional interventions, such as a diet rich in amylose corn starch to restore mucosal tolerance to allergens and modify the incidence of food allergies. The intake of such a diet can restore proper Treg cell function and contribute to increased intestinal integrity [4].

In addition to the previously described actions, SCFAs can also reduce the levels of Th2 cytokines and antigen-specific IgE, as well as inhibit reactions. In addition, they can bind to G-protein-coupled receptors and induce IL-18 production. Lower faecal SCFA levels have been shown in children with food allergies. In such individuals, the integrity of the mucosa is destroyed. The consequence of such a condition is the induction of inflammatory reactions by food antigens, with the epithelial barrier mediating their effects [22].

### 3.3. Mutual Proportions of Short-Chain Fatty Acids

The most common SCFAs in the body are butyric acid, acetic, and propionic acids. They can account for more than 95% of the total SCFA pool [37]. In the colon, the level of SCFAs is maintained at 60–150 mmol/kg. However, the proportions of acetate, propionate, and butyric acid can be represented by a ratio of 60:25:15, respectively [37]. Under normal conditions in the large intestine, acetate is present at a concentration of about 40 mM, and propionate and butyric acid at a concentration of about 15 mM [4]. In portal vein blood, acetate, propionate, and butyric acid occur in a ratio of 9:1:1 [10]. The bacterium *Faecalibacterium prausnitzii* is one of the main butyric acid producers in the human gut microbiome [38]. The ratio of SCFAs may change, at least in part, due to pH, which, at 5.5, increases the number of *Firmicutes*-type bacteria to 20% producing butyric acid [22]. Formic acid makes up a smaller part of the SCFA pool. Its exact function in the gut is not yet known, but studies to date have noted its increased concentration with inflammation [38].

### 3.4. SCFA Levels and Food Allergies

Studies to date have shown an association between SCFA levels and the increasing prevalence of food allergies. The composition and metabolic activity of the microbiota of people with food allergies differ from those of individuals without them, and lower concentrations of SCFAs have been reported in patients with food allergies [38]. Such links include the relationship between butyric acid producers, which are Clostridium spp., and their protective effect against food allergies [4]. In children whose faecal contents were examined at 1 year of age, a correlation was noted between high amounts of butyric acid and propionate and a lower risk of having a food allergy or asthma later in life. Children affected by both IgE-dependent and IgE-independent food allergies to cow’s milk were characterised by dysbiosis and reduced faecal butyric acid levels [21]. In contrast, sensitised infants at 12 months of age had lower levels of total SCFAs but also lower formic, succinic, isobutyric, valerian, and caproic acid levels. In children with food allergies, the concentrations of acetic and succinic acids were especially low. In contrast, SCFA concentrations in breast milk were not associated with sensitisation or allergies in the offspring. However, it was noted that mothers who were affected by an allergic condition had higher concentrations of several SCFAs compared to mothers with allergies [3]. In children who had a faecal test, higher levels of butyric acid correlated with a lower incidence of diagnosis of food allergies or rhinitis of allergic origin [33]. In infancy, more specifically in the third month of life, an association between the presence of mucus in the stool and 3,4-methylmalonic acid was noted [39]. This condition may be related to the activation of not only inflammatory processes but also the immune system [40].

**Figure 2:**
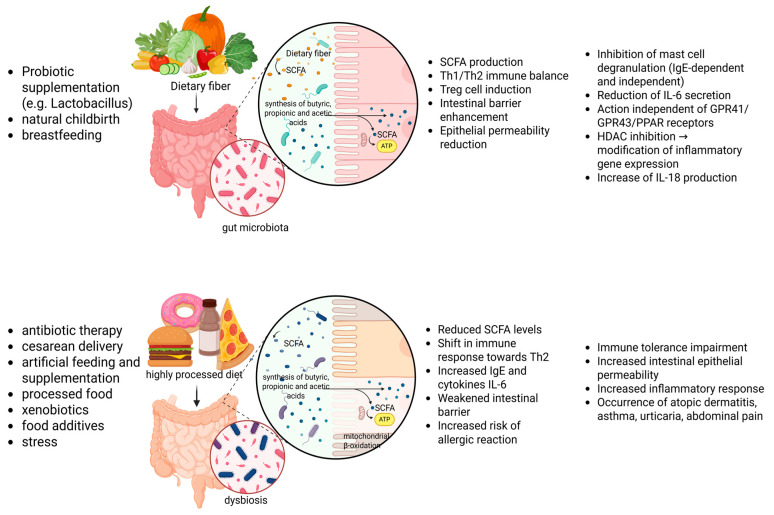
Factors influencing changes in the microbiota and its metabolites in the allergy response.

A complementary study in the field was carried out by Van Esch et al. [19]. Using a mouse model, they found that the intake of a synbiotic diet causes characteristic changes affecting the composition and activity of the microbiome. Increased total levels of SCFAs and propionic acid in particular are then observed. In the gut, the producers are predominantly *Bacteroidetes* and selected *Firmicutes*, but *Bacterium bifidobreve M-16V* may also provide an outlet for the theory of the protective effect of a synbiotic diet. In a simulation carried out under clinical conditions, allergy symptoms were reduced. A change in the gut microbiota induced by a given synbiotic diet results in an inhibition of the allergic reaction occurring due to a food allergy in a mouse model [20]. For better understanding, a figure was added to the data analysis (Figure 2).

### 3.5. Results from Animal Model Studies

A 2018 article authored by M. Andreassen et al. reports a study conducted on an animal model, specifically a mouse, on a food allergy to lupin (5.7 mg/kg bw/day) lupin protein extract) [18]. The aim of their study was to analyse the relationship between the composition of the gut microbiota, the levels of SCFAs in the gut, and sensitisation and the occurrence of anaphylaxis in mice with a food allergy to lupin. The results showed that in supernates from ileal samples, the levels of various SCFAs were mostly below the detection limit. In contrast, within the cecal and large intestinal segments, the levels of individual SCFAs were in a similar range. A difference was evident in the composition of SCFAs between the cecum and large bowel segments. One difference was the ratio of acetic to butyric acid. In the cecum segment, acetic acid, isobutyric acid, and isovaleric acid contents were higher in immunised mice than in control mice. In the study presented only the cecal segment showed significant differences in the levels and composition of short-chain fatty acids [19].

In the study, Van Esch et al. (2016) examined mice with an egg white allergy [20]. They randomly divided the mice participating in the study into five groups, each with eight female mice. They received a control diet and were sensitised to the stomach using a blunt needle with 20 mg of ovalbumin (OVA) in 0.5 mL of PBS, with 10 µg of cholera toxin used as an adjuvant. Cow-milk-free material was used to formulate the prebiotic diet. The indigestible oligosaccharides were a 9:1 mixture of short-chain fructooligosaccharides and long-chain fructooligosaccharides. The probiotic strain *B.breve* was also used. On this basis, a control diet, scFOSlcFOS diet, and B.breveiscFOSlcFOS + *B.breve* diet were formulated. The results of this study showed that in the group of mice fed scFOSlcFOS + *B.breve*, there was a reduction in allergic symptoms, as well as an increase in total SCFA levels [40]. The studies discussed are presented in the table below (Table 1).

### 3.6. Results from Human Studies

Ho HE et al. (2021) aimed to determine the oral microbiological, metabolic, and immunological profiles of individuals affected by a food allergy to peanuts [40]. This study was conducted on 105 individuals, whose unstimulated saliva was examined. When SCFA levels in saliva were examined, lower levels of acetic, butyric acids, and propionic acid were detected in the saliva of peanut-allergic subjects. The median level of acetate was 153,000 ng/mL in peanut-allergic people, compared to 229,000 ng/mL in the control group. Butyric acid, another SCFA, had a median level of 2930 ng/mL, compared to 5530 ng/mL in the control group. The last to be tested was propionate, with a median of 35,500 ng/mL, compared to 49,900 ng/mL in the control group. The results showed that people with a peanut allergy have lower levels of SCFAs in saliva and a less diverse oral saliva microbiome, but also an increased abundance of *Neisseria* spp., which may account for the effect on the metabolic and immune environments of the mucosa of people with a peanut allergy [40].

Goldberg MR et al. (2020) looked at the role of the gut microbiome in the development of IgE-dependent food allergies [38]. They recruited 291 people for the study, including 233 food allergy sufferers, from the same centre at the Institute of Allergy, Immunology and Paediatric Pulmonology, Shamir Medical Center. They included 66 people with a milk allergy, 38 people with a sesame allergy, and 71 people with a peanut or tree nut allergy for a total of 58 people. The remaining 58 people without a food allergy constituted the control group. They collected stool samples for the study. Stool SCFAs were analysed in 84 people with a single allergy, and the control group consisted of 31 people. The results of the study showed that people with a food allergy had lower concentrations of acetate, butyric, and propionic acids in their stools compared to the control group. The gut microbiota differed between food-allergic and non-allergic people [38].

Another study showing the relationship between allergy and SCFA levels is that conducted by Yu ZD et al. (2024), who aimed to trace the change in gut microbiota and SCFA levels in children affected by a cow milk protein allergy [4]. The study was conducted with 50 infants, of which 25 were allergic and 25 were controls. Stool samples were analysed in the study. The results showed that the samples from the children with a cow milk protein allergy had a lower content of SCFAs, especially acetic acid, butyric acid, and isovaleric acid. In addition, the microbial composition of the gut showed a lower abundance of *Firmicutes*, Clostridiales, and *Bacteroidetes* and a higher abundance of Sphingomonadaceae, Clostridiaceae_1, and Mycoplasmataceae than those in the control group. Infants affected by a food allergy showed lower levels of SCFAs and a different intestinal microflora structure [4].

A study by Gio-Batta M et al. in 2022 was performed to link the pattern of faecal SCFAs in children at 1 year of age to allergy prevalence at 13 years of age [1]. Stool samples from 110 individuals were examined. Allergic symptoms were associated with foods such as fruit (*n* = 17), tree nuts (*n* = 10), peanuts (*n* = 6), milk (*n* = 5), and eggs (*n* = 4). The results showed that at 1 year of age, the valerian acid concentration was inversely correlated with the development of a food allergy. The proportion of valeric acid to total SCFA concentration at 1 year of age was inversely correlated with the incidence of an allergy at 13 years of age. At 13 years of age, propionic acid concentrations were inversely related to the presence of a nut or peanut allergy, and butyric acid levels were associated with the presence of a peanut allergy. Isocaproic acid levels were not statistically significant but were associated with allergy symptoms at 1 year and 13 years. Milk allergy symptoms were inversely correlated with the proportion of isobutyric acid [1]. The following table presents an analysis of the studies performed (Table 2).

## 4. Discussion

The findings of this study contribute to the growing body of evidence linking short-chain fatty acids (SCFAs) to the modulation of immune function and the risk of food allergies. Based on the available data, two groups of factors influencing SCFA levels can be distinguished. One comprises factors that contribute to an increase in SCFA levels, which is mediated by, among other things, enrichment of the diversity of the gut microbiome. The second group consists of factors contributing to dysbiosis, which is associated with lower SCFA levels. In addition, the presence of generally lower SCFA levels has been linked to the occurrence of allergies. In infants, the occurrence of mucus in the faeces may indicate the activation of the immune system. The literature review presented the results obtained in two types of subjects. The specific SCFAs, particularly butyrate and propionate, may exert protective effects by enhancing intestinal barrier integrity, regulating Treg/Th1/Th2 balance, and suppressing pro-inflammatory cytokine responses. The first series of studies were conducted with humans. The results of the three studies presented (Ho et al., 2021 [20], Goldberg et al., 2020 [21], Yu et al., 2024 [22]) were consistent, despite the diversity of the study material (stool samples or saliva). Food-allergic subjects showed lower levels of acetate, propionic, and butyric acids than controls. SCFAs have been linked to gastrointestinal symptoms in infants, such as flatulence, intestinal gas, mucus, and constipation [40]. In addition, the study groups consisted of people with food allergies to various ingredients, such as peanuts, sesame, or cow milk protein. The results obtained suggest a link between lower SCFA levels and the occurrence of food allergies, as well as a link to changes in the diversity of the gut microbiota and saliva in food-allergic subjects. In contrast to the studies in humans, the results of studies conducted on mouse models were inconclusive. The difference observed in this study may be due to several factors. One is the temporal difference, as SCFAs in this study were assessed after the onset of the allergic response, whereas in humans, it was assessed at different stages. Another factor could have been the interspecies differences in metabolism between mice and humans. Mice are herbivores, which causes them to have a different microbiota than omnivorous humans. In consequence, the results of studies on the composition of the microbiota and its products may differ from those in humans. Low dietary fibre intake may promote the risk of food allergies by altering the gut microbiome and its metabolites SCFA [41] and BSCFA [40]. The importance of early-life microbial exposures and dietary interventions in shaping SCFA production seem obvious. While current evidence is insufficient to recommend specific supplementation strategies, modulation of SCFA levels through prebiotic or dietary fibre intake appears to be a promising preventive approach against the development of food allergies.

The limited number of studies conducted in humans and the inconsistent results may be a limitation of this study. Large-scale studies in humans, taking into account diverse backgrounds and economic and living conditions, may be required.

## 5. Conclusions

Short-chain fatty acids can be used as an indicator of microbiota disorders in humans. SCFA levels can be measured in saliva or faecal samples, providing an easy and non-invasive way to test patients. An association between SCFAs and the occurrence of food allergies has been established; people with food allergies have lower levels of butyric acid, propionic acid, acetic acid, and isovaleric acid in the stool. Factors contributing to increased SCFA levels include the diet, environment, breastfeeding, and prebiotic supplementation. Butyric acids produced by *Faecalibacterium prausnitzii* or *Clostridium* spp. plays a role in alleviating allergy symptoms, as a correlation was observed between higher levels and a reduction in the incidence of allergies. In the third month of life, a correlation was seen between 3,4-methylvaleric acid and the presence of mucus in the stool, which is an indicator of immune system activation. Further research should focus on increasing the size of the study population, taking into account ethnic, social and living factors, and identifying factors directly influencing the level of 3,4-methylvaleric acid.

## Figures and Tables

**Table 1:** SCFA levels in animals with food allergies.

Author, Year	Animals	Study Design	Tested Material	Observed Effect
M Andreassen et al., 2018 [18]	24 mice (13 were immunised)	The SCFA content in the gastrointestinal tract of lupine-sensitised mice in various sections of the intestine	Blood, stool, and tissue samples from the distal part of the small intestine, the cecum, and the colon	Mice that were immunised had higher concentrations of acetic, iso-butyric, and iso-valeric acids in the cecal segment of the intestine.
Van Esch BC. et al., 2016 [19]	40 mice	The effect of diet on egg white allergy: control diet: allergic and non-allergic group scFOSlcFOS diet, B.breveiscFOSlc-FOS diet	Blood serum samples, spleen cells	The use of the scFOSIcFOS + *B.breve* diet resulted in an increase in total SCFAs, a reduction in allergic symptoms to minor allergic shocks, activation of Th1 cells in the spleen, and a decrease in mast cell degranulation with a tendency to decrease OVA-IgE.

SCFA—short-chain fatty acids; scFOSlcFOS diet—fructooligosaccharides and long-chain fructooligosaccharides, B.breveiscFOSlc-FOS diet—fructooligosaccharides and long-chain fructooligosaccharides and probiotic; Th1 cells—type of lymphocytes.

**Table 2:** SCFA levels in patients with food allergies.

Author, Year	Number	Tested Material	Observed Effect
Ho He et al., 2021 [20]	105 people: 56 with peanut allergies and 49 healthy	Saliva	Lower levels of SCFA in the saliva of people with allergies, particularly butyric and propionic acids.
Goldberg et al., 2020 [21]	66 people with a milk allergy, 38 people with a sesame allergy, 71 people with a peanut allergy, 58 people with a tree nut allergy; 58 in the control group- healthy	Faeces	Individuals with food allergies had lower concentrations of acetic, butyric, and propionic acids compared to the control group. Significant differences in acetate concentrations were found between the group without allergies and groups with allergies to peanuts, sesame, and tree nuts. The level of butyric acid was lower in the milk and peanut allergy groups. However, the group with a peanut allergy had the lowest concentration of propionate.
Yu Z. et al., 2024 [22]	50 infants: 25 with allergies (cow milk protein allergy) 25 in the control group	Faeces	Lower SCFAs, particularly acetic, butyric, and isovaleric acid, in the group with cow milk protein allergy.
Gio-Batta et al., 2022 [1]	110 people -1 year of age with allergy at 13 years of age	Faeces	At the age of 1, the concentration of valeric acid was inversely correlated with the occurrence of food allergies to peanuts, tree nuts, fruits, milk, and eggs at the age of 13.

SCFA—short-chain fatty acids.

## Data Availability

Not applicable.

## Abbreviations

The following abbreviations are used in this manuscript:

FOS	fructooligosaccharides
GCPRs	G-protein-coupled receptors
GOS	galactooligosaccharides
SCFAs	short-chain fatty acids
